# Factors associated to acceptable treatment adherence among children with chronic kidney disease in Guatemala

**DOI:** 10.1371/journal.pone.0186644

**Published:** 2017-10-16

**Authors:** Brooke M. Ramay, Alejandro Cerón, Luis Pablo Méndez-Alburez, Randall Lou-Meda

**Affiliations:** 1 Department of Pharmaceutical Chemistry, Universidad del Valle de Guatemala, Guatemala City, Guatemala; 2 Department of Anthropology, University of Denver, Denver, Colorado, United States of America; 3 Fundación para el Niño Enfermo Renal—FUNDANIER, Hospital Roosevelt, Guatemala City, Guatemala; Postgraduate Medical Institute, INDIA

## Abstract

Pediatric patients with Chronic Kidney Disease face several barriers to medication adherence that, if addressed, may improve clinical care outcomes. A cross sectional questionnaire was administered in the Foundation for Children with Kidney Disease (FUNDANIER, Guatemala City) from September of 2015 to April of 2016 to identify the predisposing factors, enabling factors and need factors related to medication adherence. Sample size was calculated using simple random sampling with a confidence level of 95%, confidence interval of 0.05 and a proportion of 87%. A total of 103 participants responded to the questionnaire (calculated sample size was 96). Independent variables were defined and described, and the bivariate relationship to dependent variables was determined using Odds Ratio. Multivariate analysis was carried out using logistic regression. The mean adherence of study population was 78% (SD 0.08, max = 96%, min = 55%). The mean adherence in transplant patients was 82% (SD 7.8, max 96%, min 63%), and the mean adherence in dialysis patients was 76% (SD 7.8 max 90%, min 55%). Adherence was positively associated to the mother’s educational level and to higher monthly household income. Together predisposing, enabling and need factors illustrate the complexities surrounding adherence in this pediatric CKD population. Public policy strategies aimed at improving access to comprehensive treatment regimens may facilitate treatment access, alleviating economic strain on caregivers and may improve adherence outcomes.

## Introduction

Chronic Kidney Disease (CKD) is a rising epidemic known to impact cardiovascular disease risk, life expectancy, overall patient quality of life, and healthcare system costs [1,2]. In most countries, CKD in adults is associated with older age, hypertension, and diabetes. Nevertheless, the etiologies of CKD in some developing countries differ. In agricultural communities in Central America, Egypt, India and Sri Lanka, for example, CKD is commonly presented in male-farmworkers without common risk factors [3]. The uncertainty surrounding the causes of CKD in these developing countries has deemed it a public health priority to identify etiologies and ways to mitigate its impact [4].

Obtaining an early diagnosis, and sustaining long-term specialized therapy are fundamental goals of CKD pediatric care. Treatment regimens address congenital anomalies of the kidney and urinary tract (CAKUT), hereditary nephropathies, and glomerulopathies [5,6]. In addition to medicinal care, psychosocial care addresses impact of the disease on growth, quality of life, and psychosocial behavior and development. Despite specialized care needs, the quality and magnitude of health systems’ readiness to treat CKD vary around the world, and between adult and pediatric patients [7]. In low to middle income countries (LMIC), CKD is often unrecognized and is diagnosed in advanced stages of the disease [8]. As kidney function gradually declines, caretaker strain increases as they take responsibility for clinical care, nutrition, adherence to medication regimens, and development of their dependents [9]. Whereas factors attributed to positive clinical outcomes are well described in the developed world, strategies leading to successful care in CKD patients in developing countries are largely understudied and present a series of barriers in achieving optimal health care results.

Treatment adherence, or the “extent to which a person´s behavior coincides with medical or health advice” contributes to clinical outcomes, and is more complex among chronically ill children [10]. Children with CKD and their caretakers must adhere not only to medication schedules, but also to interventions such as dialysis and nutritional regimens. The prevalence of unacceptable adherence in pediatric kidney transplant patients in developed countries can be as low as 30% and as high as 70% [11]. Physiological factors, family dynamics, and illnesses all effect adherence rates in children with chronic diseases [12]., Examples in the developed world point to several factors that effect adherence and clinical outcomes of ESRD patients. Depression, anger, behavioral problems, family structure/relationships, and caretaker stress all effect adherence [13–15]. As pediatric patients go from childhood to adolescence, responsibilities of taking medications shift to the maturing patient and adherence may waver as the patient troubleshoots support systems and ways to manage their chronic illness [15]. In the LMIC setting however, factors contributing to unacceptable adherence are often overlooked and focus is shifted to the economic burden of the disease, access to health care services, and access to medications [16]. The gaps in knowledge surrounding the importance of adherence in LMIC reveal the need to intentionally address barriers to medication adherence to improve clinical outcomes.

In Guatemala, the Foundation for Children with Kidney Disease (FUNDANIER, Guatemala City) was founded in the largest tertiary-level public hospital in the country and attends patients with CKD (stage 1 through stage 5 renal disease). Patients who are diagnosed with stage 5 disease according to the Kidney Disease Outcomes Quality Initiative (KDOQI) [17] receive Hemodialysis (HD), Peritoneal dialysis (PD) or renal transplant. The program at FUNDANIER represents the country’s only comprehensive program for pediatric patients receiving HD and PD and renal transplants in Guatemala. Although many health system barriers most likely negatively affect access to medical care at FUNDANIER, the clinic provides clinical care, social support, and key medications (immunosuppressive medications, erythropoietin, activated vitamin D) to patients with CKD [18]. Nevertheless, the health care disparities faced by pediatric patients with CKD in LMIC pose challenges specific to this population, specifically in medication adherence. Identifying barriers to medication adherence that are relevant to the socio-cultural context of the patients and the clinic may help improve clinical outcomes [19].

This study aimed to identify factors associated to acceptable medication adherence among pediatric patients with stage 5 CKD. Upon identifying characteristics effecting adherence, development of clinical, and administrative policies are suggested aimed at improving clinical outcomes. Although these findings are specific to the clinical setting in Guatemala, they may be relevant to countries and clinics in similar situations.

## Materials and methods

### Study design

We carried out a cross-sectional survey aimed at determining the prevalence of acceptable treatment adherence and its associated factors. The independent variables were defined using two complementary conceptual models. First, “the behavioral model of health services use” categorizes predisposing, enabling and need factors [19,20]. Second, the “pediatric self-management model” identifies non-modifiable, and modifiable factors, as well as processes, and behaviors effecting the child, and the child´s family [10]. Independent variables are presented in S1 Table and were used to design a questionnaire that also considered factors that are known to affect access to health care in Guatemala [21–23].

### Population and sample

The study was conducted in pediatric patients with stage 5 CKD who attend FUNDANIER in Guatemala City, Guatemala. We used simple random sampling (alpha 0.05), with an expected proportion of acceptable treatment adherence of 0.82 based on a similar study in a similar sample of pediatric patients with chronic conditions in Guatemala [22]. Based on a population of 156 patients with stage 5 CKD according to FUNDANIER’s database at the end of July 2015, we calculated a sample size of 96. Inclusion criteria were stage 5 CKD as classified by FUNDANIER based on KDOQI guidelines, accompaniment by a parent or guardian, and consenting to participate.

### Data collection

We administered a questionnaire to participants with stage 5 CKD and their respective guardians. The questionnaire was administered in FUNDANIER from September of 2015 until April of 2016. Questionnaire completion lasted between 20 and 30 minutes. Participants meeting the inclusion criteria were invited to participate in the study after their normal clinic appointments. Written informed consent was carried out with guardians accompanying minors who participated in this study. Written informed assent was carried out with minors 7 years or older who participated in the study. Interviews, consent, and assent were carried out in a room separate from the clinic, assuring privacy and comfort.

### Questionnaire

The questionnaire was designed to identify the overall level of adherence and its associated factors. The adherence portion of the questionnaire posed 20 self-assessment questions with Likert scale responses based on a questionnaire originally designed and validated in HIV patients in Spain and Peru, addressing the psychosocial barriers, facilitators, and modulating factors associated to adherence [24,25]. More recently, this adherence questionnaire was adapted, applied, and validated in pediatric, HIV patients in Guatemala [22]. In order to adapt the questionnaire for use in this CKD pediatric patient population in Guatemala, the research team reviewed the questions for relevance, and then validated the questions for comprehension. The questionnaire was then validated in five patients with stage 4 CKD attending FUNDANIER to determine comprehension, internal consistency, and duration of questionnaire. The questionnaire is presented in S1 File and S2 File. The overall adherence to treatment index was worth a total of 89 points, the higher the score, the better the adherence. Adherence was calculated based on values recorded from adherence questionnaire and were classified as “acceptable” based on the conventions defined by FUNDANIER. At this clinic, renal transplant patients are classified as having “acceptable adherence” if their adherence is 95% or greater (representing 85 points out of 89 points in the current questionnaire). HD and PD participants are classified as having “acceptable” adherence if their adherence is 80% or greater (representing 71 points out of 89 points in the current questionnaire).

In addition to the cross-sectional survey, we carried out a chart review to identify the etiology of each patient with stage 5 CKD and the treatment modality they were receiving (transplant, hemodialysis, peritoneal dialysis).

### Data analysis

Descriptive statistics were used. Bivariate analysis was carried out by first classifying the overall adherence as “acceptable adherence” (95% or above for transplant patients, 80% or above for HD and PD patients), or “unacceptable adherence.” Then, independent-variable responses were transformed into “dummy” variables (also known as indicator variables or binary variables). Odds Ratio (OR) was estimated for each one of the independent variables in relation to acceptable adherence, and were measured for the entire sample (transplant, HD and PD), for each group separately (transplant patients and for HD/PD patients together). Multivariate logistic regression was carried out using relevant variables based on results of bivariate analysis and on the conceptual model. The R Project for Statistical Computing software, “R” was used to carry out all statistical analysis [26].

### Research ethics

The research conducted in this study was approved by the Institutional Review Board at the University of Denver, and the ad-hoc research ethics committee at FUNDANIER. All research was conducted according to the principles expressed in the Declaration of Helsinki.

## Results

We recruited every patient who came, until we completed 110% of the estimated sample size, that is 105 patients. Recruitment was based on predetermined clinic appointments for stage 5 CKD patients.

A total of 103 participants responded to the adherence portion of the questionnaire. Two patients declined to participate, presumably for lack of time; one was an 8-year-old, female, PD patient accompanied by her mother, and the second one was an unaccompanied 17-year-old, male, HD patient. In 52% of cases (n = 55), minors responded alone after parental consent, in 2% of cases (n = 2) guardians and minors responded together, while in 43% of cases (n = 46) guardians responded alone.

### Overall adherence

The mean adherence of the entire sample (Transplant, HD and PD patients combined) was 78% (SD 0.08, max = 96%, min = 55%). The mean adherence in transplant participants (transplant alone) was 82% (SD 7.8, max 96%, min 63%), and the mean adherence in HD and PD participants (combined) was 76% (SD 7.8 max 90%, min 55%). Based on FUNDANIER’s clinical criteria, 31% (n = 31) of the total sample had acceptable adherence, 19% (n = 7) of participants with transplant and 37% (n = 24) participants with HD and PD had acceptable adherence. Adherence questions and mean values for individual responses are listed in S2 Table.

### Predisposing factors

The mean age of the pediatric sample was 13.5 years old (SD 3.16), approximately half of the sample was female (56, 53%). Forty respondents self-identified their ethnicity as indigenous (39%), 50 respondents self-identified their ethnicity as Ladino (local term for non-indigenous or mixed race, 48%), and 13 participants chose to not identify their ethnicity (13%). The majority of participants had a primary school education (68, 66%) and just over half of the sample attended school in 2015 (61, 59%). The majority of patients’ mothers had a primary school education as their highest level of education (53, 51%), however, the educational level ranged from illiterate (10, 10%), to university level education (5, 5%). The majority of participants (68, 66%) reported living in a city at the time the questionnaire was administered, while 24% of the sample lived in small villages (n = 25), or a small town (10, 10%). Twenty-two percent of the sample reported moving due to the patients’ illness (n = 23) and reported that the child began showing symptoms of the disease when they lived in a city (15, 65%), or in a small village (7, 30%). Descriptive statistics are presented in S3 Table.

### Enabling factors

The majority of participants spoke Spanish exclusively (96, 93%), only 5 participants reported speaking both Spanish and an indigenous language (5%). Most of the participants were cared for by their mothers (74, 72%). Participants’ mothers mostly spoke Spanish (87, 84%), while eleven mothers reported speaking both Spanish and an indigenous language (11%). The range of household monthly incomes was distributed with the greatest proportion of the sample earning between $200 and $670 (47, 45%), followed by 28 participants whose household income was between $80 and $200 (27%). Eleven participants had a household income of more than $671 (11%). Upon obtaining a diagnosis, it took patients an average of 364 days to be referred to FUNDANIER for treatment (SD 869, min = 0, max = 14 years). On average patients travelled for 2.5 hours to arrive to clinic visits at FUNDANIER (SD 1.6, min = 10 min, max = 8hrs). The majority of respondents and their families arrived to clinic appointments by bus (68, 66%), the only means of public transportation in Guatemala, while a smaller proportion of the sample arrived by a combination of transport methods (18, 17%) and their own vehicles (car or motorcycle) (14, 14%).

### Need factors

Of the sample, 38% of participants were receiving Peritoneal Dialysis (PD) (n = 39), 25% were receiving Hemodialysis (HD) (n = 25), and 35% were renal transplant patients (n = 36) at the time the questionnaire was administered. The etiologies of these stage 5 CKD patients were mainly CKD of an undetermined cause (75, 73%), followed by miscellaneous causes (13, 13%), congenital abnormalities (5, 5%), and glomerulopathies (5, 5%). Only twenty participants presented comorbidities (19%) that ranged from cardiac, to respiratory and immunological illnesses. Forty percent of participants did not know what caused their illness (n = 41), 16% believed their illness was a result of their diet (n = 16), and 10% believed it was due to genetic causes (n = 10). These results can be found in S3 Table. The database containing the original results can be found in S1 Dataset.

### Bivariate analysis

Bivariate analysis of the entire sample revealed that patients had higher and statistically significant odds of acceptable adherence when the mother had a technical school degree, and when the monthly income was greater than $667. Patients had lower and statistically significant odds of acceptable adherence when the monthly household income was between $80–200. Several factors regarding the time it takes to get to the clinic and current residence of geographic location affected the odds ratio, but these results were not statistically significant. See Table 1 for a summary of results with p-value less than 0.4.

**Table 1:** Bivariate analysis for HD, PD and transplant participants combined.

	Independent variable	Odds ratio	p-value
Enabling Factors	Arrives to clinic visits in a personally owned vehicle	2.22	0.22
	Arrives to clinic visits in a public bus	0.57	0.23
	Monthly income between $201–666	1.68	0.27
	Monthly income between $80–200	*0.2	0.01
	Monthly income greater than $667	*4.43	0.03
	Patients´ mother speaks Spanish and an indigenous language	0.49	0.38
	Patients´ mother speaks Spanish only	2.33	0.28
	Takes 2 to less than 3 hours to arrive to FUNDANIER clinic visits	0.62	0.39
	Takes 3 to less than 4 hours to arrive to FUNDANIER clinic visits	2.11	0.16
	Takes 5 to less than 6 hours to arrive to FUNDANIER clinic visits	0.25	0.15
Need Factors	Complication or emergency due to renal disease in 2015	0.43	0.06
Predisposing Factors	Mother´s educational level: illiterate	1.98	0.38
	Mother´s educational level: primary school education incomplete	0.48	0.11
	Mother´s educational level: some secondary school education	0.53	0.36
	Mother´s educational level: technical school	*3.46	0.03
	Other neighbors have renal disease	2.12	0.11
	Resides in the Central region of Guatemala	0.57	0.31
	Resides in the Highlands of Guatemala	2.84	0.20
	Resides in the Metropolitan region of Guatemala	1.67	0.25
	Sex of patient (female)	1.67	0.25
	Sex of patient (male)	0.6	0.25
	The family has moved households due to renal illness	1.77	0.30

Table 1 includes results with a p value less than 0.4

*p<0.05

Bivariate, subgroup analysis of patients receiving HD and PD revealed that two enabling factors significantly affect adherence in this population (p<0.05). Patients whose mothers speak Spanish-only had higher odds of acceptable adherence (OR = 6.43, p = 0.05), and patients whose family members had a monthly income between $80–200 had lower odds of acceptable adherence (OR = 0.19, p = 0.01).

Subgroup analysis of patients with transplant resulted in no significant odds ratio associations. Nevertheless, those reporting an income greater than $667 and presence of other community members with renal disease had higher odds of acceptable adherence (p = 0.06).

### Multivariate analysis

Logistic regression was carried out using variables based on the conceptual model and the bivariate analysis, privileging those variables that showed statistical significance (see Table 2). Significant predisposing factors positively effecting adherence were mothers’ higher level of education, and current residence in the metropolitan area. Mothers with a high school/technical school degree and mothers with university degree, each had CKD-dependents with better adherence.

**Table 2:** Multivariate analysis for entire sample using logistic regression.

	Andersen´s category (or type of factor)	Estimate	Std. Error	p value
**Predisposing Factors**	Patient sex	1.14097	0.74802	1.525
	Indigenous ethnicity	-1.16751	0.75842	-1.539
	Patient attended school in 2015	0.20366	0.7926	0.257
	Mother completed high school/technical school	3.68731	1.27124	2.901**
	Mother completed university	3.78103	1.69229	2.234*
	Current residence metropolitan area	1.97474	0.89096	2.216*
**Enabling Factors**	Monthly income Less than $80	-0.93111	1.19402	-0.78
	Mother cares for patient	-0.93111	1.19402	-0.78
	Takes less than 3 hours to arrive to clinic appointments	-1.06269	0.8662	-1.23
**Need Factors**	Etiology of an undetermined cause	0.90236	0.94845	0.951
	Presence of a concomitant disease	-0.64225	0.79194	-0.811
	Perceived cause of disease: doesn't know	-0.07096	0.65875	-0.108

** p<<0.05

* p<0.05

## Discussion

One third of patients (HD, PD and transplant patients combined) involved in this study had acceptable adherence. These findings are consistent with findings in other countries [12]. Although transplant patients showed better overall adherence compared to HD and PD patients, only 19% achieved “acceptable adherence” given a higher requirement for adherence goals to maintain immunosuppressive levels. One systematic review presenting 36 papers from 1990 to 2008 found that 30% of renal transplant pediatric patients had unacceptable adherence [27]. Unacceptable adherence has also been documented in children with other chronic diseases such as HIV and liver transplant [28,29]. Nevertheless, patients with CKD face unique difficulties in treatment adherence. Self-esteem and self-appearance during adolescence and puberty are problematic as CKD progresses revealing their effect on growth contributing to anxiety and denial, all of which negatively affect adherence [12,15]. One study showed that patients, similar to this study’s participant demographic, experience similar day-to-day challenges that are compounded with extremely limited resources resulting in malnutrition, disadvantages in caretaker-employment, and household/environmental sanitation [6]. Identifying factors associated to treatment adherence presents a means to support patients in achieving improved adherence and improved clinical outcomes.

Families in lower income categories in this study had higher odds of poor overall adherence, pointing to the economic strain placed on caregivers of children with CKD. Treatment related cost, cost of medications and time lost at work all contribute to financial losses and have been attributed to decreased quality of life, higher incidence of comorbidities, and reduced clinical outcomes in caregivers of patients with CKD [30]. The financial burden of disease has been similarly reported in adult patients with CKD in the United States where direct healthcare costs have shown to increase with disease progression [31]. Health related costs such as transportation, lodging and meals add to nonmedical costs for family members taking care of patients traveling long distances to seek health care related services. In pediatric patients, the caretakers of children with CKD jeopardize stable employment in order to take care of their child and are faced with significant medical and non-medical costs eventually contributing to disease progression [32]. The sample of respondents in this study with lower income had higher odds of having unacceptable adherence, those with higher income had higher odds of having acceptable adherence.

Participants in the study were more likely to have acceptable adherence if their mothers had a high school/technical degree. In Guatemala 1 of every 10 residents is illiterate, and females receive an average of 10.5 years of schooling, one of the lowest rates in the Central American region [33,34]. A high school/technical degree in Guatemala represents a relatively high level of education and characterizes the environment surrounding the child, and may even shed light on the socio-economic situation of the household. When the mother is also the caretaker of the child (as is the case for 72% of participants in this study), her educational level also serves as a proxy for the level of care a patient receives in the household [35]. The mother’s educational level in this study may reflect a household environment that may enhance the mother’s capacity to manage their child’s disease, either as a direct caretaker, or as a contributor to the household, or both. In the long run, children with CKD depend highly on their caregivers requiring very specific, intensive support from those who raise them [30]. A higher level of education may contribute to their efficiency. The World Health Organization (WHO) has shown that completing higher levels of formal education results in higher incomes, better employment opportunities, overall improved living conditions and have all been linked to better health outcomes [36]. The connection between the level of formal education and adherence however is not always clear. In one review of post-transplant patients, educational level predicted adherence rates in adults, but had no effect in pediatric patients [27]. In the present study, the mother’s higher level of education reflects improved pediatric CKD adherence.

Although this study was not designed to detect differences in ethnicity and adherence, findings suggest that participants whose mothers spoke Spanish-only had higher odds of acceptable adherence in comparison to their peers whose mothers spoke an indigenous language in addition to Spanish. Differences in race (not ethnicity) have been shown to effect adherence, exemplifying the effects of social inequalities on medication outcomes in patients living in their country of origin, or as immigrants in foreign countries [37]. Barriers to access and utilization of health care facilities for indigenous peoples have been documented in the Guatemalan health care system, although not specifically for chronic kidney disease [33,38,39]. Findings from this study may be used to explore how language and ethnicity impact the levels of acceptable adherence in this population and others in the surrounding region.

This study showed unacceptable adherence in 78% of participants, which was shown to be associated to financial, educational and linguistic characteristics of participants and their households. Factors associated with unacceptable adherence, such as lower income, mother’s lower education level, and perhaps mother’s ethnicity may be used for developing targeted interventions that combine educational and social work support. Additionally, the findings highlight the importance of developing administrative and financial interventions aimed at improving the availability and affordability of medicines in the LMIC setting, by addressing the barriers to accessibility [37]. Findings from the WHO indicate that these small steps in education and workforce placement as well as in access to medications may be useful to improve adherence outcomes.

Multivariate analysis carried out in this study revealed that participant residence in the metropolitan area had a small but significant positive effect on adherence. Nevertheless, bivariate analysis regarding distance to clinic, and current residence, were non-significant. Further studies need to be carried out to confirm causalities in the multivariate analysis in order to provide recommendations regarding the distance to the clinic and the impact it may have on adherence posing specific questions related to access to health care services and access to medications. Non-modifiable challenges in adherence such as economic status may be addressed through exploring specific barriers in access to medication in Guatemala. Collaboration between the Ministry of Health (MOH), and international organizations facilitating medication access may be a useful strategy to provide medications to pediatric patients with CKD. As medication access is facilitated, clinical care outcomes may become more achievable for CKD patients across socio-economic sectors receiving healthcare in Guatemala.

## Conclusions

Reported adherence was lower than expected at FUNDANIER, but is consistent with CKD clinics in other countries. Factors that all positively impacted adherence were: higher educational level of patient´s mother, higher reported income, and living in metropolitan area. This paper reinforces themes that are generalizable across resource limited countries including the economic burden of disease, access to healthcare services, and access to medications drawing attention to complexities faced in health care in underdeveloped countries. Given the effect of socio-economic status on adherence, the WHO recommends taking small steps in educational interventions to improve adherence outcomes [37]. Although these types of interventions are complex in design and implementation, a series of workshops combined with support groups aimed at parents and caregivers of Transplant, HD and PD patients may improve overall adherence in patients seeking care at FUNDANIER and in clinics in similar socio-economic settings. Additionally, improving strategic policies aimed at strengthening public health institutions and autonomy of renal treatment facilities may contribute to preventing disease and improving access to renal replacement therapy thereby alleviating economic strain on caregivers that may improve adherence outcomes. Limitations of this study include those inherent to the cross-sectional research design and self-reported questionnaires.

## Supporting information

S1 Dataset Adherence Fundanier. (CSV)

S1 File Study questionnaire Spanish. (PDF)

S2 File Study questionnaire English. (DOCX)

S1 Table Variables presumed to effect pediatric adherence. The independent variables were defined using “the behavioral model of health services use” and the “pediatric self-management model”.
(DOCX)

S2 Table Adherence questions, scores of allowed responses, and mean results. (DOCX)

S3 Table Socio demographic data of stage 5 CKD pediatric participants. (DOCX)
