# Supplementary material for: Factors associated to acceptable treatment adherence among children with chronic kidney disease in Guatemala
Source: PLoS One. 2017 Oct 16;12(10):e0186644. doi: 10.1371/journal.pone.0186644 (PMC5643062; doi:10.1371/journal.pone.0186644)
Supplement: S1 File — (PDF) [file pone.0186644.s002.pdf]

Elegibilidad

1. Numero del expediente en FUNDANIER

text: ________________________________

2. Codigo del participante

integer: ________________________________

3. Estadio del paciente

integer: ________________________________

4. ¿Dispuesto a participar?

select one:

- sí
- no
- nr

5. ¿Tiene diagnostico de ERC en Julio 2015?

select one:

- sí
- no
- nr

Cuestionario

"4. ¿Dispuesto a participar?" = si
and
"5. ¿Tiene diagnostico de ERC en Julio 2015?" = si

Seccion A Datos Generales

6. Encuestador

select one:

- Luis Pablo
- Brooke
- Alejandro

7. El paciente esta respondiendo a la encuesta?

select one:

- sí
- no
- nr

8. ¿Cual es su relación con [nombre del niño/a]?

“7. El paciente esta respondiendo a la encuesta?” = no

select one:

- madre
- padre
- hermano/a
- otro familiar
- otro

9. Sexo de la persona respondiendo a la encuesta

“7. El paciente esta respondiendo a la encuesta?” = no

select one:

- femenino
- masculino

10. Edad de la person respondiendo a la encuesta

“7. El paciente esta respondiendo a la encuesta?” = no

integer: ________________________________

Seccion B Predisposing Factors

11. Edad en años cumplidos del niño/a:

integer: ________________________________

12. Sexo del niño/a:

select one:

- femenino
- masculino

13. ¿Ustedes se consideran indígenas o no?

select one:

- sí
- no
- nr

14. ¿Cual es el ultimo grado que el ninio/a ha aprobado en la escuela?

select one:

- primero primaria
- segundo primaria
- tercero primaria
- cuarto primaria
- quinto primaria
- sexto primaria
- primero basico
- segundo basico
- tercero basico
- diversificado
- no responde
- ninguno

15.¿Asiste el nino/a a la escuela durante 2015?

select one:

- si
- no
- irregularmente
- no responde

16.¿Cual es el grado de escolaridad de la mama del nino/a?

select one:

- analfabeta
- primaria incompleta
- primaria completa
- secundaria incompleta
- secundaria completa
- estudios de nivel diversificado
- estudios de nivel universitario
- nr

17.¿Actualmente, en qué municipio y departamento viven?

cascading select: (long list)

18. ¿Actualmente, vive en una ciudad, pueblo, aldea o paraje?

select one:

- ciudad
- pueblo
- aldea
- paraje
- nr

19.¿Cuál es el nombre del lugar donde vive? Si vive en ciudad, incluir en que zona vive.

text: ________________________________

20.¿Se ha cambiado de casa desde que empezó la enfermedad?

select one:

- si
- no
- irregularmente
- no responde

Preguntas B Pq Cambio De Casa

"20.¿Se ha cambiado de casa desde que empezó la enfermedad?" = si

21. ¿por qué?

text: ________________________________

22. ¿Cuándo empezó a enfermarse el niño/a, en que municipio y departamento vivían?

select one:

- sí
- no
- nr

23. ¿Cuándo empezó enfermarse el niño/a, vivía en una ciudad, pueblo, aldea o paraje?

select one:

- ciudad
- pueblo
- aldea
- paraje
- nr

24. ¿Cuál es el nombre del lugar donde vivía cuando empezó a enfermarse el niño/a? Si vivía en una ciudad, incluir en que zona vivía.

text: ________________________________

25.¿Hay otras personas con la misma enfermedad de los riniones en su colonia/aldea/pueblo?

select one:

- si
- no
- irregularmente
- no responde

26. ¿En la colonia, aldea o pueblo?

"25.¿Hay otras personas con la misma enfermedad de los riniones en su colonia/aldea/pueblo?" = si

select multiple:

- colonia
- aldea
- pueblo
- nr

27. ¿Cuantas personas son?

"25.¿Hay otras personas con la misma enfermedad de los riniones en su colonia/aldea/pueblo?" = si

integer: ________________________________

28. Segun usted ¿que cree que provocó que (a su hijo/a) le diera esta enfermedad?

audio: ________________________________

Seccion C Enabling Factors

29.¿Cuales son los ingresos mensuales aproximadamente en su hogar (incluyendo lo que generan diferentes personas)?

select one:

- menos de Q600
- Q600-Q1500
- Q1500-5000
- mas de Q5000
- nr

30. ¿Qué idiomas habla el niño/a?

select one:

- solo castellano
- solo idioma indigena
- castellano e idioma indígena
- nr

31.¿Qué idiomas habla la madre del niño/a?

select one:

- solo castellano
- solo idioma indigena
- castellano e idioma indígena
- nr

32. ¿Quién es la persona que principalmente se encarga de cuidar la salud del niño/a?

select one:

- madre
- padre
- hermano
- otro familiar
- otro
- turnos de varios
- nr

33.¿Cuántas horas se tarda en llegar desde su casa a FUNDANIER a la hora de su cita?

decimal: ________________________________

34.¿Qué medio de transporte usa normalmente para llegar a sus citas a FUNDANIER?

select one:

- buses del transporte publico
- taxi
- vehiculo propio
- vehiculo alquilado
- combinacion
- otros
- nr

35.¿Aproximadamente cuántos días pasaron desde que el niño/a empezo a enfermarse hasta que llegó por primera vez a FUNDANIER?

integer: ________________________________

36.¿Aproximadamente cuántos días pasaron desde que el niño/a llegó por primera vez a FUNDANIER y le dijeron a usted exactamente lo que tenía?

integer: ________________________________

Seccion D Adherencia

37.El entrevistado sabe de los medicamentos y el entrevistado responde a las preguntas de adherencia?

select one:

- sí
- no
- nr

38.El nino tiene mas de 7 años y responde a las preguntas de adherencia

select one:

- sí
- no
- nr

Preguntas D Adherencia

"37.El entrevistado sabe de los medicamentos y el entrevistado responde a las preguntas de adherencia?" = si
or
"38.El nino tiene mas de 7 años y responde a las preguntas de adherencia" = si

39. Si en algún momento se sentía mejor el niño u observó mejor al niño, ¿dejó de administrar el medicamento?

select one:

- siempre
- más de la mitad de las veces
- la mitad de las veces
- alguna vez
- ninguna ocasión
- nr

40. Si en algún momento se sentía muy mal usted u observó que el niño se sentía mal ¿dejó de tomar/dar el medicamento?

select one:

- siempre
- más de la mitad de las veces
- la mitad de las veces
- alguna vez

- ninguna ocasión
- nr

41. Si en algún momento se sentía triste o deprimido usted/el niño ¿dejó de tomar/dar el medicamento?

select one:

- siempre
- más de la mitad de las veces
- la mitad de las veces
- alguna vez
- ninguna ocasión
- nr

42. De los medicamentos que tiene que tomar, cuantos toma siempre?

select one:

- ninguno
- algunos
- todos
- nr

43. ¿Cómo calificaría la relación que tiene con el médico que lo/los ha atendido?

select one:

- Mala
- Algo mala
- Regular
- Buena
- Excelente
- nr

44. ¿Cuánto esfuerzo le causa mantener el tratamiento, venir a sus citas, a usted/con el niño en términos de adherencia? (dieta, puntual a citas, seguir indicaciones, economía, distancia de clínica)

select one:

- Mucho
- Bastante
- Regular
- Poco
- Nada
- nr

45. ¿Cómo evalúa la información que ha recibido sobre los medicamentos? (considera que le han dado suficiente información sobre la medicina)

select one:

- Nada
- Poco
- Regular
- Bastante
- Mucho
- nr

46. ¿Qué tan beneficioso considera usted que es el uso de estos medicamentos?

select one:

- Nada
- Poco
- Regular
- Bastante
- Mucho
- nr

47. ¿Considera que la salud de usted/el niño ha mejorado desde que empezó a utilizar los medicamentos?

select one:

- Nada
- Poco
- Regular
- Bastante
- Mucho
- nr

48. ¿Ha dejado de tomar el medicamento el niño/usted?

select one:

- siempre
- más de la mitad de las veces
- la mitad de las veces
- alguna vez
- ninguna ocasión
- nr

49. ¿Se siente capaz de seguir/apoyar (al niño) con el tratamiento/ tomando el tratamiento?

select one:

- Nada
- Poco
- Regular
- Bastante
- Mucho
- nr

50. ¿Suele tomar/dar los medicamentos a la hora correcta?

select one:

- Nunca
- Algunas veces
- La mitad de las veces
- Muchas veces
- Siempre
- nr

51. Cuando los resultados de los análisis son buenos ¿suele su médico utilizarlos para darle ánimos y seguir adelante con los medicamentos?

select one:

- Nunca
- Algunas veces
- La mitad de las veces
- Muchas veces
- Siempre
- nr

52. En general, ¿cómo es el grado de satisfacción (se siente feliz) de usted/del niño desde que empezó a tomar su tratamiento?

select one:

- Muy insatisfecho
- Insatisfecho
- Ambivalente
- Satisfecho
- Muy satisfecho
- nr

53. ¿Cómo valoraría la intensidad de los efectos secundarios relacionados con la toma de los medicamentos?

select one:

- Muy intensos
- Intensos
- Medianamente intensos
- Poco intensos
- Nada intensos
- nr

54. ¿Cuánto tiempo cree que ocupa en tomar/administrar los medicamentos? (cuanto tarda, esperares que vómita, forzar a tomar)

select one:

- Mucho
- Bastante

- Regular
- Poco
- Nada
- nr

55. Considera que para administrar/ dar los medicamentos ud es una persona adherente (Adherencia – cumple con dosis, hora, pastillas, etc)?

select one:

- Nada cumplidor
- Poco cumplidor
- Regular
- Bastante cumplidor
- Muy cumplidor
- nr

56. ¿Cuánta dificultad percibe usted al momento de administrar el medicamento? (tamaño de tableta, sabor)

select one:

- Mucho
- Bastante
- Regular
- Poco
- Nada
- nr

57. Desde que usted/el niño está en tratamiento ¿alguna vez ha dejado de administrarle/tomar sus medicamentos un día completo o más?

select one:

- sí
- no
- nr

58. ¿Utiliza alguna estrategia para acordarse de administrar/tomar los medicamentos

select one:

- sí
- no
- nr

Seccion E Utilizacion De Servicios

59. En lo que va de 2015, ¿cuántas citas ha tenido con el doctor en la clínica de FUNDANIER?

integer: ______________________________

60. En lo que va de 2015, ¿cuántas citas ha tenido para dar muestras de laboratorio?

integer: ______________________________

61. En lo que va de 2015, ¿cuántas citas ha tenido para diálisis u otros procedimientos?

integer: ______________________________

62. Entonces, en lo que va de 2015, ¿cuántas citas ha tenido en total?

integer: ______________________________

63. ¿Ha faltado alguna cita?

select one:

- si
- no
- irregularmente
- no responde

64. ¿A cuantas citas ha faltado?

"63. ¿Ha faltado alguna cita?" = si

integer: ______________________________

65. ¿Cuales son los motivos de haber faltado?

"63. ¿Ha faltado alguna cita?" = si

text: ______________________________

66.¿En lo que va de 2015, ha necesitado que lo atiendan en FUNDANIER antes de su cita?

select one:

- sí
- no
- nr

67.¿Cuántas veces?

"66.¿En lo que va de 2015, ha necesitado que lo atiendan en FUNDANIER antes de su cita?" = si

text: ______________________________

68. ¿Por qué motivo?

"66.¿En lo que va de 2015, ha necesitado que lo atiendan en FUNDANIER antes de su cita?" = si

text: ______________________________

69.¿Lo atendieron?

"66.¿En lo que va de 2015, ha necesitado que lo atiendan en FUNDANIER antes de su cita?" = si

select one:

- sí
- no
- nr

70.Porque no lo atendieron?

"69.¿Lo atendieron?" = no

text: ________________________________

Seccion F Need Factors

71. ¿El nino/a tiene otras enfermedades ademas de la pura enfermedad de los rinones?

select one:

- si
- no
- irregularmente
- no responde

72. ¿Cuáles?

"71. ¿El nino/a tiene otras enfermedades ademas de la pura enfermedad de los rinones?" = si

text: ________________________________

73.¿El niño/a ha tenido en 2015 alguna complicación o emergencia por causa de le enfermedad de los riñones?

select one:

- si
- no
- irregularmente
- no responde

74.¿Cuáles?

"73.¿El niño/a ha tenido en 2015 alguna complicación o emergencia por causa de le enfermedad de los riñones?" = si

text: ________________________________
