# Supplementary material for: Factors associated to acceptable treatment adherence among children with chronic kidney disease in Guatemala
Source: PLoS One. 2017 Oct 16;12(10):e0186644. doi: 10.1371/journal.pone.0186644 (PMC5643062; doi:10.1371/journal.pone.0186644)
Supplement: S2 File — (DOCX) [file pone.0186644.s003.docx]

**Questionnaire Adherence in CKD children in Guatemala:**

ELIGIBILITY

1. Number of the record in FUNDANIER

Number

1. Participant Code

Randomly generated code

1. ESRD Stage

Number

1. Is the person willing to participate?

Yes, no-END

1. Does the child have a diagnosis of ESRD in 2015?

Yes, no-END

**QUESTIONAIRE**

SECTION A GENERAL INFORMATION

1. Indicate who is conducting the interview

Indicate by first name

1. Is the child responding to the questionnaire?

Yes, no

1. What is your relationship with [child's name]?

Father, mother, brother, another family member, other

1. Indicate the sex of the person responding to the questionnaire

Male, Female

1. Age of the person responding to the survey

Whole number in years

SECTION B PREDISPOSING FACTORS

1. How old is the child

Whole number in years

1. Indicate the sex of the child

Male, female

1. Do you identify yourself as indigenous?

 Yes No No response

1. What is the highest grade that the child has completed in school?

No education, Secondary school, Primary school, Highschool/technical school

1. Has the child attended school in 2015?

Yes, no, no response,

1. What is the educational level of the mother of the child?

Illiterate , Primary school, Secondary school, Highschool/Technical school. University, No response

1. Currently, in which municipality (county) and department (state) do you live?

Select the GeographicID

1. Do you currently live in a city, town, village or hamlet (smaller than a village)?

City, ​​town, village, smaller than village, No response

1. Indicate the name of the city and zone where you live

Text

1. Have had to change houses (or where you live) because of ESRD?

Yes, No, Maybe, No response

1. Why?

Text

1. When the child got sick, in which municipality or department did you live?

Select the GeographicID

1. When the child got sick, did you live in a city, town, village or hamlet (smaller than village)?

City, ​​town, village, smaller than village, No response

1. What is the name of the place where the child lived when she/he began to get sick child?

 Text

1. Are there others with the same disease in your neighborhood / village / town?

Yes, No, No response

1. Specify: Are the others with the same disease in the neighborhood, village or town?

Neighborhood, village, town, No response

1. How many people are there in your neighborhood, village or town with the disease?

Whole number

1. In your opinion what do you think caused that (your son / daughter) get this disease?

Field worker listens to response and records a field worker-voice note summary of what is said

**SECTION C ENABLING FACTORS**

1. What is the approximate monthly income in your household (including what generate different people)?

Less than $80, Between $80-200, Between $201-670, More than $671, No response

1. What language does the child speak?

Spanish, Spanish and indigenous language, Indigenous language only, No response

1. What languages are ​​spoken by the child’s mother?

Spanish, Spanish and indigenous language, Indigenous language only, No response

1. Who is the person primarily responsible for the health of the child?

Mother, Father, Rotate between various caretakers, A brother or sister, Other, No Response

1. How many hours it takes to get from home to FUNDANIER at the time of your appointment?

Whole number

1. What means of transportation do you normally use to get to their appointments FUNDANIER?

Bus, A combination of transport methods, Family owned vehicle (car or motorcycle), Taxi, No response

1. How many months passed from when the child began to first get sick until they first came to FUNDANIER?

Whole number

1. How many days passed since the child first came to FUNDANIER and a diagnosis was given?

Whole number

SECTION D ADHERENCE

1. Does the respondent know enough about the child`s care to respond to questions about medicines and adherence?

Yes, No, No response

1. Is the child is over 7 years old capable of answering adherence questions? (informed assent completed in Q9)

Yes, No, No response

ADHERENCE QUESTIONS

1. If in any moment you observed the patient feeling better, did you stop giving the patient their medication (or did they stop taking the medication)?

Always, Sometimes, Not much, Never

1. If in any moment the patient felt sick, did you stop giving the patient their medication (or did they stop taking the medication)?

Always, Sometimes, Not much, Never

1. If in any moment you observed the patient feeling sad, did you stop giving them the medication (or did they stop taking the medication)?

Always, Sometimes, Not much, Never

1. Of all of the medications you take, how many do you take all the time?

None, not all, some, all

1. How would you rate the relationship you have with the doctor and the health care team?

Poor, fair, good, excellent

1. Do you think you have a sufficient amount of information regarding the medication the patient uses for ESRD?

Insufficient, not enough, enough, more than enough

1. How hard is it for you to maintain your treatment adherence, and come to your appointments for ESRD?

Very hard, hard, not hard, not hard at all

1. In your opinion, how beneficial is taking these medications?

Not at all, Not, Beneficial, Very beneficial

1. Do you think that the patient´s health has improved since you started giving them medication for ESRD?

Not at all, not much, some, very much

1. Has the patient stopped taking their medication at any time?

Always, Sometimes, Not much, Never

1. Do you feel capable supporting the patient in taking their medication to treat their illness (or do you feel capable taking medication for your illness)?

Not at all, not much, some, very much

1. Do you give the patient the medications at the same time every day (or does the patient take their medication at the same time every day)?

Never, Not much, Sometimes, Always

1. When you receive good news about the progress of your disease does your doctor use the news to encourage you to continue taking your medication?

Never, Not much, Sometimes, Always

1. In general, how happy are you (and the patient) since the patient started taking their medication for ESRD?

Very unsatisfied, Dissatisfied, Neutral, Satisfied

1. How do you rate the intensity of the side effects experienced related to these medications?

Very intense, Intense, Neutral, Not intense

1. How much time do you spend taking medications for ESRD?

A lot of time, it takes some time, normal amount of time, not much time at all

1. Do you consider yourself adherent to the patients´ medication therapy (or your medication therapy)?

Never, Not much, Sometimes, Always

1. How difficult do you perceive taking medication for ESRD?

Very hard, hard, not hard, not hard at all

1. Since the patient began medication therapy for ESRD, have they ever missed a complete day of taking their medications?

Yes, No

1. Do you or the patient use any sort of strategy to remember to take their medications?

Yes, No

SECTION E HEALTH UTILIZATION SERVICES

1. So far in 2015, how many appointments have you had with the doctor at the clinic FUNDANIER?

Whole number

1. So far in 2015, how many appointments have you had to give laboratory samples?

Whole number

1. So far in 2015, how many appointments have you had dialysis or other procedures?

Whole number

1. Then, in 2015 so far, how many appointments have you had in total?

Whole number

1. Have you missed any of these appointments?

Yes, no

1. How many of these appointments have you missed?

Whole number

1. What are the reasons for having missed appointments?

Text

1. So far in 2015, have you needed to be cared for in FUNDANIER before your appointment?

Yes, No, No response

1. How many times?

Whole number

1. For what reason?

Text

1. Were you seen?

Yes, No, No response

1. Why not?

Text

SECTION F NEED FACTORS

1. Does the child have other diseases besides the kidney disease?

Yes, No, No response

1. What disease?

Text

1. Has the child in 2015 had any complications or emergencies because of her kidney disease?

Yes, No, No response

1. What complications or emergencies?

Text
