# Supplementary material for: Factors associated to acceptable treatment adherence among children with chronic kidney disease in Guatemala
Source: PLoS One. 2017 Oct 16;12(10):e0186644. doi: 10.1371/journal.pone.0186644 (PMC5643062; doi:10.1371/journal.pone.0186644)
Supplement: S1 Table — The independent variables were defined using “the behavioral model of health services use” and the “pediatric self-management model”. (DOCX) [file pone.0186644.s004.docx]

**S1 Table. Variables presumed to effect pediatric adherence**

| Predisposing factors | Patient age  Patient sex  Ethnicity  Educational level of patient  Current attendance in school  Educational level of the mother  Current residence  If family changed residence due to illness  Where patient lived when they first demonstrated symptoms |
| --- | --- |
| Enabling factors | Monthly income  Language spoken by patient  Language spoken by mother  Primary caregiver  How long it takes to arrive to the clinic  Mode of transportation |
| Need factors | Etiology  Treatment modality  Presence of comorbidity  Perceived cause of the disease |
