# Supplementary material for: Factors associated to acceptable treatment adherence among children with chronic kidney disease in Guatemala
Source: PLoS One. 2017 Oct 16;12(10):e0186644. doi: 10.1371/journal.pone.0186644 (PMC5643062; doi:10.1371/journal.pone.0186644)
Supplement: S2 Table — (DOCX) [file pone.0186644.s005.docx]

**S2 Table. Adherence questions, scores of allowed responses, and mean results**

| Question | Likert scale range of responses | Mean (±SD) |
| --- | --- | --- |
| If in any moment you observed the patient feeling sad, did you stop giving them the medication (or did they stop taking the medication)? | Always=1, Never=5 | 4.63(±0.64) |
| If in any moment the patient felt sick, did you stop giving the patient their medication (or did they stop taking the medication)? | Always=1, Never=5 | 4.59(±0.67) |
| Do you feel capable supporting the patient in taking their medication to treat their illness (or do you feel capable taking medication for your illness)? | Not at all=1, Very much=5 | 4.54(±0.56) |
| If in any moment you observed the patient feeling better, did you stop giving the patient their medication (or did they stop taking the medication)? | Always=1, Never=5 | 4.49(±0.74) |
| Has the patient stopped taking their medication at any time? | Always=1, Never=5 | 4.32(±0.77) |
| How would you rate the relationship you have with the doctor and the health care team? | Poor=1, Excellent=5 | 4.22(±0.80) |
| Do you give the patient the medications at the same time every day (or does the patient take their medication at the same time every day)? | Never=1, Always=5 | 4.00(±0.76) |
| In your opinion, how beneficial is taking these medications? | Not at all=1, Very much=5 | 3.93(±0.60) |
| Do you consider yourself adherent to the patients´ medication therapy (or your medication therapy)? | Never=1, Always=5 | 3.89(±0.82) |
| In general, how happy are you (and the patient) since the patient started taking their medication for ESRD? | Unsatisfied=1, Satisfied= 5 | 3.88(±0.81) |
| How do you rate the intensity of the side effects experienced related to these medications? | Very intense=1, Not intense at all=5 | 3.88(±0.90) |
| When you receive good news about the progress of your disease does your doctor use the news to encourage you to continue taking your medication? | Never=1, Always=5 | 3.85(±0.85) |
| How much time do you spend taking medications for ESRD? | A lot=1, Not much=5 | 3.82(±0.83) |
| Do you think that the patient´s health has improved since you started giving them medication for ESRD? | Not at all=1, Very much=5 | 3.82(±0.64) |
| How difficult do you perceive taking medication for ESRD? | Very hard=1, Not hard at all=5 | 3.79(±0.88) |
| Do you think you have a sufficient amount of information regarding the medication the patient uses for ESRD? | Insufficient=1, More than enough=5 | 3.74(±0.67) |
| How hard is it for you to maintain your treatment adherence, and come to your appointments for ESRD? | Very hard=1, Not hard at all=5 | 1.90(±0.86 |
| Of all of the medications you take, how many do you take all the time? | None=0, All=2 | 1.60(±0.49) |
| Since the patient began medication therapy for ESRD, have they ever missed a complete day of taking their medications? | yes=0, no=1 | 0.90(±0.30) |
| Do you or the patient use any sort of strategy to remember to take their medications? | yes=0, no=1 | 0.53(±0.50) |
