# Supplementary material for: Factors associated to acceptable treatment adherence among children with chronic kidney disease in Guatemala
Source: PLoS One. 2017 Oct 16;12(10):e0186644. doi: 10.1371/journal.pone.0186644 (PMC5643062; doi:10.1371/journal.pone.0186644)
Supplement: S3 Table — (DOCX) [file pone.0186644.s006.docx]

S3. Table. Socio demographic data of pediatric participants (n=103)

| **Pre-disposing factors** | **Patient age (years)** | Frequency | % |
| --- | --- | --- | --- |
|  | 5-9 | 13 | 12% |
|  | 10-14 | 44 | 43% |
|  | 15-18 | 46 | 45% |
|  | **Patient sex** |  |  |
|  | Female | 55 | 53% |
|  | Male | 48 | 47% |
|  | **Patient ethnicity** |  |  |
|  | Indigenous | 40 | 39% |
|  | Ladino | 50 | 48% |
|  | No response | 13 | 13% |
|  | **Patient attended school in 2015** |  |  |
|  | Yes | 61 | 59% |
|  | No | 41 | 40% |
|  | Irregularly | 1 | 1% |
|  | **Educational level of patients' mother** |  |  |
|  | Illiterate | 10 | 10% |
|  | Primary school | 53 | 51% |
|  | Secondary school | 15 | 15 |
|  | High School/Technical school | 15 | 15% |
|  | University | 5 | 5% |
|  | No Response | 5 | 5% |
| Enabling factors | **Patient language** |  |  |
|  | Spanish | 96 | 93% |
|  | Spanish and indigenous language | 5 | 5% |
|  | No response | 2 | 2% |
|  | **Language spoken by mother** |  |  |
|  | Spanish | 87 | 84% |
|  | Spanish and indigenous language | 11 | 11% |
|  | Indigenous language only | 1 | 1% |
|  | No response | 4 | 4% |
|  | **Patient caregiver** |  |  |
|  | Mother | 74 | 72% |
|  | Father | 3 | 3% |
|  | Rotate between various caretakers | 11 | 10% |
|  | A brother or sister | 4 | 4% |
|  | Other | 10 | 10% |
|  | No Response | 1 | 1% |
|  | **Monthly Income** |  |  |
|  | Less than $80 | 9 | 9% |
|  | Between $80-200 | 28 | 27% |
|  | Between $201-670 | 47 | 45% |
|  | More than $671 | 11 | 11% |
|  | No Response | 8 | 8% |
| Need factors | **Treatment** |  |  |
|  | Peritoneal dialysis | 39 | 38% |
|  | Hemodialysis | 26 | 25% |
|  | Transplant | 36 | 35% |
|  | Unknown | 2 | 2% |
|  | **Etiology** |  |  |
|  | CKD-Undetermined cause | 75 | 73% |
|  | Congenital abnormalities (CAKUT) | 5 | 5% |
|  | Glomerulopathies | 5 | 5% |
|  | Hereditary nephropathies | 2 | 2% |
|  | Miscellaneous causes | 13 | 12% |
|  | Uknown | 3 | 3% |
